# Electroplastic Cyclic Deformation of CuZn30 Brass

**DOI:** 10.3390/ma19102119

**Published:** 2026-05-18

**Authors:** Wojciech Weiler, Karol Jaśkiewicz, Zbigniew Zimniak

**Affiliations:** Metal Forming, Welding and Metrology Department, Wrocław University of Science and Technology, ul. Ignacego Łukasiewicza 5, 50-371 Wrocław, Poland

**Keywords:** plastometric tests, cyclic torsion, electrically assisted, electroplastic effect, CuZn30 brass

## Abstract

This article presents the results of research on electrically assisted forming (EAF) in the process of cyclic oscillatory torsion of CuZn30 brass. Experiments were conducted using pulsed electric current with varying parameters: pulse durations of 0.5, 2.5, and 5 ms, and pulse intervals ranging from 0.5 to 30 ms. Reference data for the electrically assisted torsion tests were obtained from conventional tests performed under identical conditions without current flow. A pronounced thermal effect was observed for specific current parameters. To accurately determine the impact of temperature rise on the deformability of CuZn30 brass during cyclic torsion, the authors conducted additional tests at elevated temperatures—corresponding to the average temperatures recorded during the EAF trials—without current application. In all investigated cases, EAF during cyclic oscillatory torsion led to a flow stress reduction ranging from nearly 8% to almost 25% compared to current-free trials. Furthermore, applying current parameters where the pulse interval exceeded the pulse duration resulted in a significant increase in strain to failure, ranging from nearly 25% up to 110% relative to the reference samples. The study also examined isophase current characteristics (where pulse duration equals pulse interval), which yielded results that clearly deviated from other configurations. The application of isophase pulses triggered a different material response, leading to a degradation of deformability by more than 21%. The presented research and findings may contribute to the further development of novel, energy-efficient, and advanced manufacturing processes in metal forming.

## 1. Introduction

EAF processes constitute a part of the broader field of electrically assisted manufacturing (EAM). This is an advanced processing technology that can lead to significant reductions in stress, improved formability, and alterations in the properties of metallic materials. EAF technologies enhance deformability by exploiting the electroplastic effect (EPE), which is induced by the flow of electric current through metals. In recent years, these innovative methods have been gaining increasing attention, resulting in numerous new research studies [1,2].

Experiments involving the application of electrical pulses are most frequently carried out in tensile tests [3,4,5,6,7,8,9,10,11,12,13], as this configuration is the simplest to perform. The electrically assisted tensile deformation of titanium alloys has been investigated in studies [3,4,5,6,7,8,9]. This method is highly effective for examining the formability of these difficult-to-shape alloys, which typically require elevated temperatures for forming. In article [3], experiments were conducted on the electrically assisted cyclic loading and unloading of Ti-6Al-4V alloy sheets. Both forced and natural cooling were applied in order to analyze the thermal and athermal effects of electric current flow. The results showed that the thermal effect of the electric current accounts for as much as 75% of the total stress reduction and plays a key role in enhancing ductility. The athermal effect emerges once a threshold current density and an appropriate level of plastic deformation are reached. It increases monotonically with current density, leading to the formation of “hot spots”, weakening of atomic bonds, intensification of local strain gradients, substructure formation, and nucleation. In article [4], the mechanism of the EPE influencing the behavior of the TA15 titanium alloy was investigated based on dislocation motion. The authors developed an EAF constitutive model that incorporates both the thermal and athermal effects of electric current flow.

In study [5], electrically assisted quasi-static tensile tests and electrically assisted tensile tests with strain-rate jump were performed at a current density of 10–20 A/mm^2^ and a specimen surface temperature of 182.7–452.3 °C. The yield strength and tensile stress decreased monotonically, and material weakening became noticeable with increasing current density. Electrically induced “hot spots” located in defect-rich regions (such as grain boundaries or phase boundaries), weakened the local strength and created nucleation sites for new grains or new phases. In article [6], electrically assisted tensile tests of commercially pure titanium were carried out at various current densities, examining the mechanical properties, strain distribution, and microstructural evolution. The results indicate that both the flow stress and the strain-hardening capability decrease with increasing current density. In study [7], tensile tests of the TC4 alloy were performed under electrically assisted conditions using high-intensity (6000 A, 9000 A) and low-frequency (1 Hz) pulsed currents at different deformation rates (19.8, 5, and 0.1 mm/min). The application of electric current reduced the stress by 15–44%. Decreasing the deformation rate to 0.1 mm/min led to an increase in elongation. The flow of high-density pulsed current modifies the structure and arrangement of dislocations, facilitating their accumulation, which promotes the formation of subgrains with dislocation localization along their boundaries. As a result, the fraction of deformed grains in the microstructure decreases by approximately 30%. In study [8], electrically assisted uniaxial tensile tests were performed on Ti65 titanium alloy foil with a thickness of 0.1 mm and a grain size ranging from 7.2 μm to 22.3 μm. The results showed that electric-current-induced weakening and hardening are strongly dependent on grain size. The initial grain size and the strain required to trigger recrystallization exhibit an inverse correlation. The volume of recrystallized areas increases with current density in fine-grained material; however, in coarse-grained material, the effect of current density on recrystallization is limited. The authors developed a constitutive model that links grain size with multiple strengthening and weakening mechanisms. In article [9], tensile tests of Grade 2 titanium were conducted both without and with electric current. It was demonstrated that increasing the intensity of the electrical pulse results in reduction in both yield strength and strain. Tensile deformation without current activates dislocation slip and twinning at relatively high tensile stress. Pulsed electric current activates dislocation slip, which leads to a reduction in tensile stress and suppresses twinning. The results indicate the possibility of achieving substantial stress reduction with only a minor decrease in elongation.

In studies [10,11], electrically assisted tensile tests of aluminum alloys were conducted. EAF increases the deformability of aluminum alloys while allowing the material to retain its mechanical properties after forming. In article [10], the effects of electrical pulse coupling, temperature, strain rate, and strain on the formability of the 7075-T6 alloy were examined. An isothermal tensile test and an electrically assisted isothermal tensile test were performed at the same temperature, and numerical simulation was additionally carried out using ABAQUS 2023. The results showed that electrical pulses reduced the flow stress and significantly increased elongation. Constitutive equations incorporating the EPE were proposed. Moreover, electrically assisted three-point bending tests were performed, and it was found that the springback angle decreases with increasing current density. In article [11], the deformation behavior of the 2024-O aluminum alloy during the EAF process was described using the Hill48 plasticity model combined with the Grosman hardening model. Relationships between the model parameters and temperature, current density, and strain rate were also investigated.

Studies [12,13,14] concern electrically assisted tensile of composite materials. In study [12], Cu–Al LC laminate composites were investigated, while article [13] examined Ti/TiC composites. The reduction in flow stress was analyzed, and the mechanism of electric-current interaction with dislocations was described, including hardening behavior associated with microstructural evolution. Based on modeling, the respective contributions of hardening and the EPE to the overall stress were calculated. A constitutive model was developed that accounts for both current-induced hardening and weakening. In article [14], composite Cu/stainless steel strips were subjected to tensile deformation. A predictive model was developed for the electrically assisted rolling of Cu/SS strip. Rolling experiments and simulation tests were subsequently performed to examine the influence of various pulsed-current conditions and deformation parameters on the behavior of the composite strip. The results indicate that increasing the pulsed-current intensity promotes deformation of the stainless steel component and enhances the compatibility between two metals during rolling.

In study [15], an electrically assisted rolling process was carried out to form micro-grooves in GH99 alloy foil. The application of electric current significantly increased the groove depth and reduced elastic deformation, while elevated temperatures improved the forming quality. The longer pulse duration and higher current density further increased the groove depth, but exceeding a certain critical level led to cracking. Modeling and simulations incorporating thermal effects were used to analyze elastic behavior. It was demonstrated that the use of electric current enhanced plasticity and reduced elasticity.

In article [16], the electrically assisted extrusion process of magnesium tubes with micro-grooves was investigated, along with the influence of mechanical and electrical parameters on dimensional accuracy, microstructure, and mechanical properties. It was found that reducing the extrusion speed and applying electric current contributed to a decrease in forming forces. Increasing the extrusion speed and current intensity leads to grain growth, reduced strength and ductility, and a shift in the fracture mechanism from mixed (ductile–brittle) to brittle. In article [17], the authors proposed an electro-thermo-mechanically coupled crystal-plasticity constitutive model for pure copper, illustrating the influence of electrical and thermal stimuli on plastic deformation. The thermal and athermal effects of electric current flow are described independently, corresponding respectively to temperature and current density.

In article [18], an electrically assisted hot-pressing process was presented for joining TA1 titanium and Q235 low-alloy steel within a temperature range of 700–850 °C under a load of 10 MPa. For comparison, a conventional diffusion bonding process with hot pressing, without electrical assistance, was also conducted. The application of electric current significantly enhanced elemental diffusion. The activation energy for TiC layer growth during electrically assisted hot pressing and conventional hot pressing was 137.37 kJ/mol and 190.29 kJ/mol, respectively. At 800 °C, the maximum shear strength of the joint produced using the EAF process was 214.5 MPa, whereas in the conventional hot-pressing process it was 139.7 MPa.

In study [19], die for electrically assisted bending of quenched low-carbon steel with strength of 1500 MPa was described. Compared with cold bending without electric current, electrically assisted bending reduced the dislocation density, which improved the material’s ductility and decreased both the forming resistance and the proportion of elastic deformation in the overall forming process. The pulsed electric current also noticeably reduced the springback angle of the sheet.

In article [20], tensile and stamping tests were performed on sheet material made from a Ti_2_AlNb-based alloy, which is known to be difficult to form. Such alloys typically consist of two or three phases (α2, B2/β, and O). The B2/β phase serves as the matrix and the primary carrier of plastic deformation, while the α2 phase acts as a strengthening phase. As temperature increases, the brittle α2 phase tends to precipitate along grain boundaries, effectively pinning them, hindering grain growth, and restricting deformation. During plastic deformation, a large number of dislocations are generated within the O phase and at O/β phase interfaces, creating additional obstacles to further deformation. Consequently, Ti_2_AlNb-based alloys exhibit limited formability, even under hot forming conditions. The study demonstrated that pulsed electric current influences the microstructural evolution: a current intensity of 50–60 A at a strain rate of 0.1–0.001 s^−1^ decreases the volume fraction of the α2/O phases and increases the fraction of the B2/β phase, thereby improving the alloy’s formability.

In study [21], an electrically assisted forging process was carried out to join two different aluminum alloys, AA6061 and AA7075. Cross-sectional analysis revealed a sound, defect-free joint. The results confirmed the suitability of this method for producing lightweight structural components made from dissimilar materials. Electrically assisted simultaneous joining and forging improves the time and energy efficiency of the process while ensuring high joint quality and enabling precise geometric forming.

In study [22], electro-plastometric torsion of cylindrical CuZn30 brass specimens was carried out for the first time. The influence of electrically assisted cyclic torsion on the stress and strain behavior of CuZn30 brass was examined. The application of electrical pulses with various parameters consistently resulted in stress reduction of 4–10% compared with tests performed without current. The stress decreased with increasing pulse duration and with shorter intervals between successive pulses. In most tests, the electric current flow led to an increase in strain relative to tests without current. The maximum strain increase reached 38%, although for certain pulse parameter settings a decrease in strain was observed.

In summary, the above review of selected recent studies demonstrates that EAF processes alter the properties of metals. Phenomena induced by electric current flow lead to improved formability, reduced stress, and accelerated microstructural evolution.

In the present article, cyclic oscillatory torsion of CuZn30 brass was investigated, taking into account the effects of temperature and the ratio of pulse duration to the interval between successive pulses on stress and strain. Particular attention was paid to the influence of current with isophase characteristics. For the first time, torsion with electric current flow—producing a specific temperature—was compared with torsion without current performed at an analogous temperature obtained by heating using a conventional method.

## 2. Experimental Equipment and Materials

### 2.1. Experimental Equipment

A proprietary, patented torsion plastometer was used for the experiments. A custom-built high-current power supply with maximum operating voltage of 2.7 V was employed to supply electric current. The system was equipped with a supercapacitor battery with total capacitance of 14,000 F. The desired pulse shape was controlled using an AXIOMET AX-DG2010AF function generator (AXIOMET, Łódź, Poland). The current intensity flowing through the specimen was measured using an RCT Rogowski coil (Power Electronic Measurements, Ltd., Long Eaton, UK) and monitored with Rigol DS1052E digital oscilloscope (Rigol, Wokingham, UK).

The high-current power supply was connected to the specimen using flexible copper cables terminated with special tips. Especially in the case of monotonic (unidirectional) torsion, applying current to the sample—which completes several or even a dozen full rotations before breaking—posed an exceptionally difficult design challenge. The authors have submitted a patent application for the power supply system, along with the design of the sample holders and the sliding swivel tips of the power supply cables. Patents pending. The nominal voltage before each test was 2.52 V. The temperature of the deforming specimen was monitored continuously.

**Figure 1:**
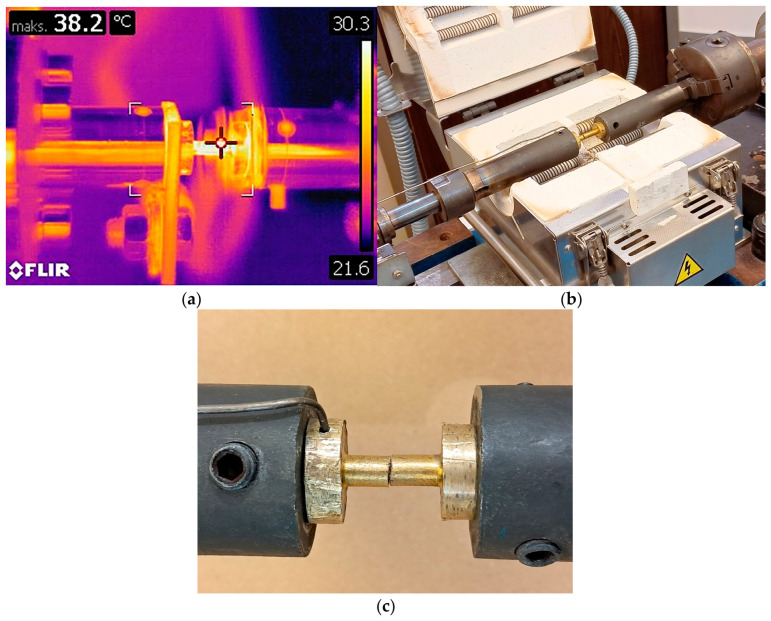
Thermal imaging camera view of the sample during electrically assisted torsion (**a**), broken sample in the electric stove (**b**) and the method of attaching the thermocouple to the sample (**c**).

In the conventional tests (without current flow) conducted at elevated temperatures, the specimen was heated in a resistance furnace, shown in Figure 1b. The temperature was monitored using a Class 1 K-type thermocouple (NiCr-Ni), visible in Figure 1c. The thermocouple was inserted into a hole in the specimen flange, drilled at a 90° angle and reaching the specimen’s axis. It was positioned from the side of the stationary grip, ensuring it did not undergo deformation during the torsion process. The thermocouple was calibrated within a range of –40 to +1000 °C. For measurements up to +375 °C, the measurement accuracy is ±1.5 °C, while in the range of +375 to +1000 °C, the accuracy is ±0.4% of the reading.

In the EAF tests, temperature was measured using a FLIR T840 thermal imaging camera (FLIR, Wilsonville, OR, USA). The measurement accuracy of the camera is ±2 °C for ranges up to 100 °C and ±2% of the reading for ranges above 100 °C. The spatial resolution of the camera with the lens used in the study is 0.90 mrad/pix. To prevent reflections that could interfere with temperature measurement, the gauge length of the specimen was coated with a matte black paint, and the camera’s emissivity was set to 0.95. Calibration was performed automatically, enabled and set by default within the camera’s software.

### 2.2. Experimental Material

The investigated CuZn30 brass was supplied in the form of extruded bars with diameter of 20 mm. The material was subjected to homogenization annealing at 700 °C for 60 min, after which cylindrical specimens were machined from the prepared stock, with a gauge section diameter of 6 mm and a gauge length of 12 mm. The specimen used in the tests is shown in Figure 1.

### 2.3. Experimental Methodology

The authors are pioneers in applying electrically assisted torsion testing within bulk metal forming processes. The aim of the study was to compare electrically assisted torsion with conventional torsion (without electric current) performed at the same temperature as that generated by current flow. Such a comparison should allow the determination of the influence of the EPE on formability during torsion.

Based on extensive earlier investigations and the results reported in [22], the following deformation variants of CuZn30 brass were selected for the present study:

- conventional cyclic (bidirectional) torsion with a strain rate of $\dot{\varepsilon }$ = 1 s^−1^ and a strain amplitude of A = 0.06, performed at room temperature as well as at 100 °C and 120 °C;
- electrically assisted cyclic (bidirectional) torsion with a strain rate of $\dot{\varepsilon }$ = 1 s^−1^ and a strain amplitude of A = 0.06, using electric current characterized by a pulse duration *t_d_* and an interval (pause) *t_p_* between successive pulses;
- conventional monotonic (unidirectional) torsion with a strain rate of $\dot{\varepsilon }$ = 1 s^−1^, performed at room temperature and at 100 °C and 120 °C;
- electrically assisted monotonic (unidirectional) torsion with a strain rate of $\dot{\varepsilon }$ = 1 s^−1^, using electric current with pulse duration *t_d_* and an interval *t_p_* = 3*t_d_* between successive pulses.

The application of higher strain rates in torsion tests causes a significant temperature increase and a highly non-uniform temperature distribution along the length of the specimen, which may lead to uncontrolled deformation. Therefore, a strain rate of $\dot{\varepsilon }$ = 1 s^−1^ was adopted for the experiments, as it does not cause a considerable temperature rise in the twisted specimen.

The applied strain amplitude A = 0.06 results from the rotation amplitude of the specimen, equal to 0.1 revolutions. Based on their previous studies, the authors concluded that large rotation amplitudes—exceeding 0.2 revolutions of the specimen—do not significantly affect the stress level compared to the results obtained under classical monotonic torsion. As indicated by the authors’ previous studies focusing primarily on brasses and bronzes [23,24], the application of complex deformation paths—including symmetric cyclic deformation [24] and cyclic deformation combined with tension or compression [23]—significantly influences the increase in strain and changes in stress levels compared to monotonic deformation results. The impact of the applied deformation methods on stress is not unequivocal. It depends on temperature, strain rate, material type, and, most crucially, the selected deformation path.

A positive effect of variable and complex deformation paths on flow stress reduction across the entire strain range is observed only at low amplitude values, generally not exceeding 0.2 of a specimen revolution. It was also noted that symmetric cyclic deformation is more advantageous than incremental cyclic torsion (i.e., involving different amplitudes in different deformation directions) [24]. In general, it can be concluded that large deformation amplitudes, significantly exceeding 0.2 revolutions, do not substantially affect the yield stress level relative to the results obtained during monotonic deformation.

In electrically assisted cyclic torsion, the following current parameters were applied: pulse durations 0.5, 2.5, and 5 ms, and pause times between successive pulses 0.5, 1, 1.5, 2.5, 5, 7.5, 15, and 30 ms. The measured current density ranged from 146.5 to 167.1 A/mm^2^. In electrically assisted monotonic torsion, pulse durations of 0.5, 2.5, and 5 ms were used, along with pulse pauses 1.5, 7.5, and 15 ms, while the current density ranged from 142.7 to 159.3 A/mm^2^.

In the torsion test, the torsional moment is measured as a function of the sample’s angle of twist, and these values are then converted into stress and strain. The precise determination of the stress–strain relationship depends on the used measurement data processing method as well as the accuracy of the deformation control system. In this study, the method applied to determine the relationship between the angle of twist and the stress and strain describes the flow stress with the following equation [22]:

(1) $$\sigma_{p}=\sqrt{3}\tau =\sqrt{3}\frac{3M(m+n)}{2\pi r_{rz}^{3}}$$

The equivalent strain is described by the equation:

(2) $$\varepsilon_{red}=\frac{1}{\sqrt{3}}\gamma =\sqrt{3}\frac{r_{rz}\omega }{l_{rz}}$$

where *τ*—shear stress,

*M*—torsional moment,

*n*—strain hardening factor,

*m*—strain rate sensitivity factor,

*ω*—specimen torsional angle,

*r_rz_*—specimen true radius,

*l_rz_*—specimen true length,

*γ*—non-dilatational strain.

The strain hardening factor *n* is defined by the following equation:

(3) $$n=\frac{N}{M}\frac{\delta M}{\delta N}$$

The strain rate sensitivity factor *m* is defined by the following equation:

(4) $$m=\frac{\dot{N}}{M}\frac{\delta M}{\delta \dot{N}}$$

where *M*—torsional moment,

*N*—number of specimen rotations,

$\dot{N}$—specimen torsion rate.

The strain hardening factor *n* can be determined directly from the *M*–*N* curve via computer-aided differentiation within the software of the plastometer used for the tests. To determine the strain rate sensitivity factor *m*, the torsional moment *M* must be established for constant values of specimen revolutions *N* and varying twisting speeds $\dot{N}$. In trials conducted at ambient temperature, the strain hardening factor *n* was 0.435, while the *m* factor was neglected due to its negligible value. At elevated temperatures, the strain rate sensitivity factor *m* was 0.202, whereas the *n* factor was neglected as its value was very low. The strain amplitude was determined using Formula (2), based on the twist angle of the specimen. For instance, for a specimen twist angle of *ω* = 180°, the strain amplitude is A = 0.3.

**Figure 2:**
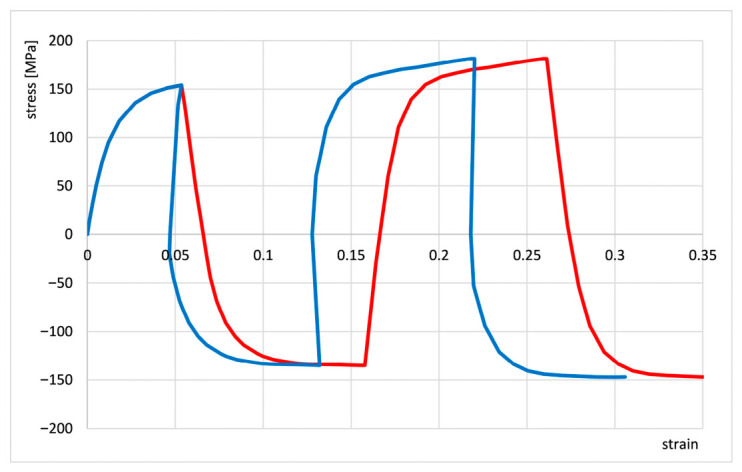
Comparison of the cyclic oscillatory torsion curve with strain amplitude A = 0.06 containing elastic strains (red line) with the true curve (corrected—blue line) [22].

In bulk forming processes, it is often assumed that elastic strains are so small compared to plastic strains that they can be neglected. However, during cyclic oscillatory torsion tests, elastic strain occurs in every deformation cycle and makes it difficult to read the plastic strain. Figure 2 compares stress curves for cyclic oscillatory torsion with a strain amplitude of 0.06 as a function of total strain (plastic strain + elastic strain, red line) and as a function of plastic strain only (blue line). The comparison shows that even for a small total strain—for example, 0.27—the share of elastic strains in a given test reaches about 20%, which leads to significant errors in the interpretation of the obtained results. With further deformation, this share increases. The authors used the method of separating elastic and plastic strains. A special computer program was implemented in the plastometer software to calculate plastic strains in individual hysteresis loops of cyclic strain [22].

Each test under given deformation conditions was repeated three times. When one of the results differed significantly from the others (over 5%), the test was repeated for verification purposes, therefore the authors did not perform statistical calculations.

## 3. Results

During the experiments involving the application of electrical pulses with duration of *t_d_* = 0.5 ms, the measurements indicated only a very small temperature increase, with the maximum temperature reaching approximately 38 °C. Since the average temperature from all tests performed with a pulse duration of *t_d_* = 0.5 ms was 35.7 °C, the results obtained with current assistance were compared to the results without current, measured at room temperature.

**Figure 3:**
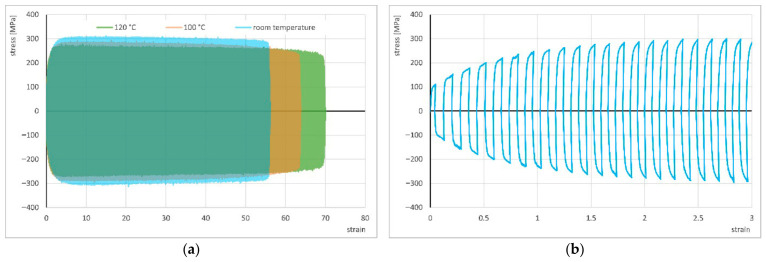
Comparison of the cyclic torsion stress–strain curves with strain rate $\dot{\varepsilon }$ = 1 s^−1^ and strain amplitude A = 0.06 at room temperature, 100 °C and 120 °C (**a**) and a close-up of the initial segment of the curve at room temperature (**b**).

In contrast, in the tests with electrical pulses of *t_d_* = 2.5 ms and 5 ms and with pause times between successive pulses of *t_p_* = 3*t_d_* or longer, a very pronounced thermal effect was observed, with average temperatures reaching 100 °C and 120 °C, respectively. Therefore, to more accurately determine the influence of temperature increase on the deformability of CuZn30 brass in cyclic torsion, the authors decided to conduct additional tests at elevated temperatures, without applying electrical pulses. The results of cyclic torsion at room temperature and at 100 °C and 120 °C, without current flow, are presented in Figure 3 and Table 1.

**Figure 4:**
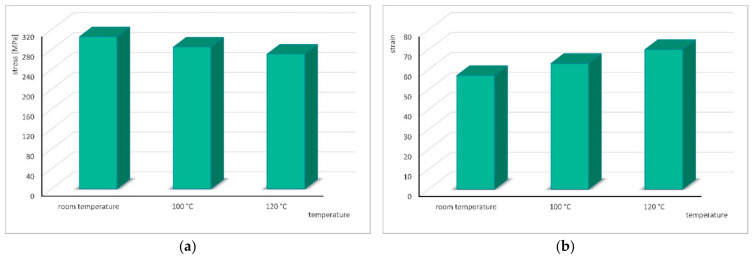
Change in stress (**a**) and strain (**b**) depending on temperature during cyclic torsion with strain rate $\dot{\varepsilon }$ = 1 s^−1^ and strain amplitude A = 0.06.

To better illustrate the obtained results, bar charts showing the variations in stress and strain as a function of temperature were prepared and are presented in Figure 4.

**Figure 5:**
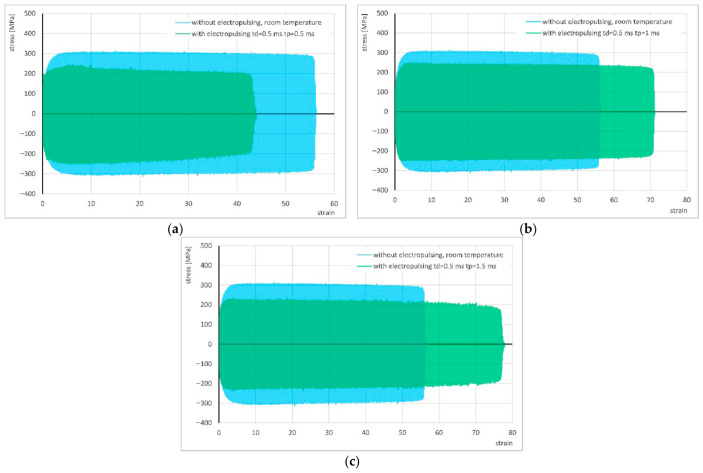
Comparison of the stress–strain curve of cyclic torsion without current flow at room temperature and the stress–strain curves using electrical pulses with duration *t_d_* = 0.5 ms and pause times *t_p_* = 0.5 ms (**a**), 1 ms (**b**) and 1.5 ms (**c**).

The results of cyclic torsion with the application of electrical pulses of *t_d_* = 0.5 ms and pause times between successive pulses of 0.5, 1, and 1.5 ms were compared with an analogous test without current application and are presented in Figure 5 and Table 2. In all electrically assisted experiments, the stress decreased very significantly—by more than 75 MPa compared to the test without current application.

The flow of pulsed current with a pulse duration of *t_d_* = 0.5 ms and a pause time of *t_p_* = 0.5 ms, i.e., under isophase conditions (pulse duration equal to the pause time, *t_d_* = *t_p_* = 0.5 ms), resulted in a reduction in strain compared to the test without electrical pulses. In the remaining two tests, with pause times between pulses two and three times longer than the pulse duration, a clear increase in strain occurred compared to the results obtained without current.

**Figure 6:**
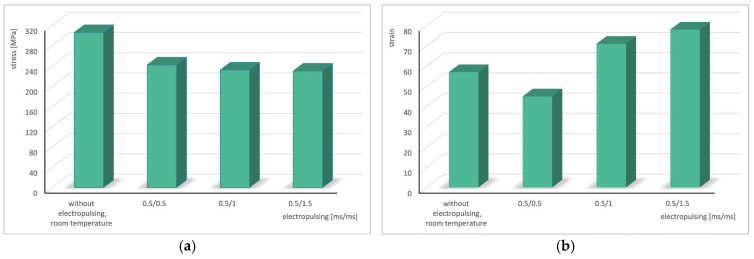
Change in stress (**a**) and strain (**b**) in the cyclic torsion test with the application of electric pulses of duration *t_d_* = 0.5 ms depending on the pause *t_p_*.

To better illustrate the obtained results, bar charts showing the variations in stress and strain as a function of the pause time between successive electrical pulses were prepared and are presented in Figure 6.

**Figure 7:**
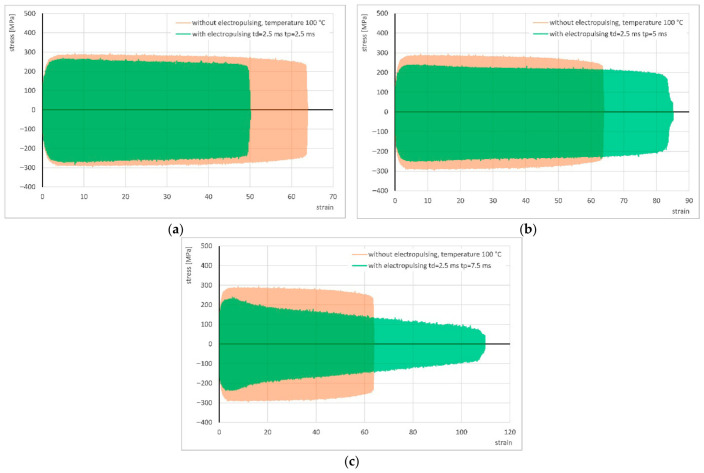
Comparison of the stress–strain curve of cyclic torsion without current flow at 100 °C and the stress–strain curves using electrical pulses with duration *t_d_* = 2.5 ms and pause times *t_p_* = 2.5 ms (**a**), 5 ms (**b**) and 7.5 ms (**c**).

The results of cyclic torsion with the application of electrical pulses of *t_d_* = 2.5 ms and pause times between successive pulses of *t_p_* = 2.5, 5, and 7.5 ms are presented in Figure 7 and Table 3, compared with the corresponding torsion results without current application, obtained at temperature of 100 °C.

In these experiments, the stress decreased by approximately 24 to 57 MPa relative to the test without current. The application of pulses with pause times of *t_p_* = 5 and 7.5 ms led to significant increase in strain compared to the tests without current flow, whereas the use of pulsed current under isophase conditions (*t_d_* = *t_p_* = 2.5 ms) once again resulted in a reduction in strain.

**Figure 8:**
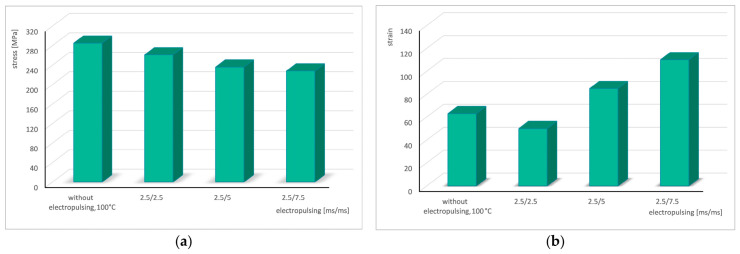
Change in stress (**a**) and strain (**b**) in the cyclic torsion test with the application of electric pulses of duration *t_d_* = 2.5 ms depending on the pause *t_p_*.

The following bar charts illustrate the variations in stress and strain as a function of the pause time between successive electrical pulses (Figure 8).

For the experiments described above (*t_d_* = 0.5 ms and *t_d_* = 2.5 ms), pulsed current was applied with pause times equal to the pulse duration, as well as with pause times two and three times longer. For the tests involving electrical pulses with duration of *t_d_* = 5 ms, the authors decided to use pulsed current under isophase conditions (*t_d_* = *t_p_* = 5 ms), as well as with a pause time of *t_p_* = 3*t_d_* = 15 ms and a significantly extended pause time of *t_p_* = 6*t_d_* = 30 ms.

**Figure 9:**
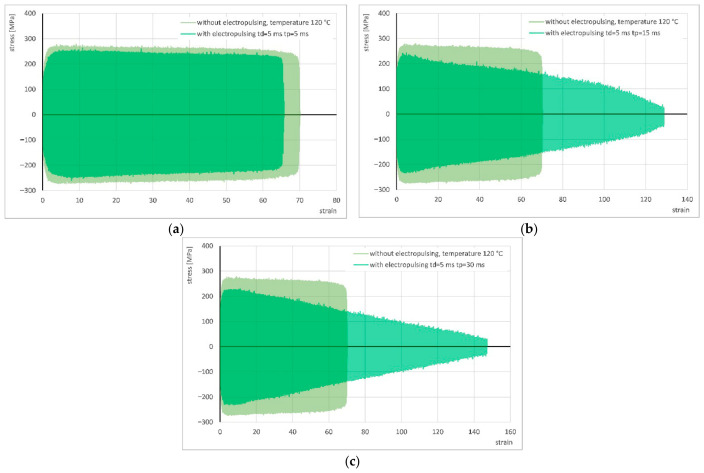
Comparison of the stress–strain curve of cyclic torsion without current flow at 120 °C and the stress–strain curves using electrical pulses with duration *t_d_* = 5 ms and pause times *t_p_* = 5 ms (**a**), 15 ms (**b**) and 30 ms (**c**).

During all experiments with an application of electrical pulses of *t_d_* = 5 ms, a significant temperature increase was again observed, with the average temperature reaching 120 °C. Figure 9 presents the stress–strain curves obtained during cyclic torsion with electrical pulses of *t_d_* = 5 ms and pause times of *t_p_* = 5, 15, and 30 ms, compared with the torsion results without current application at temperature of 120 °C. The flow of pulsed current caused a reduction in stress ranging from approximately 21 MPa to about 48 MPa. The isophase current condition (*t_d_* = *t_p_* = 5 ms) once again produced a deterioration in strain, whereas the application of pause times *t_p_* = 15 ms and *t_p_* = 30 ms resulted in very substantial increases in strain. A summary of the results is provided in Table 4.

**Figure 10:**
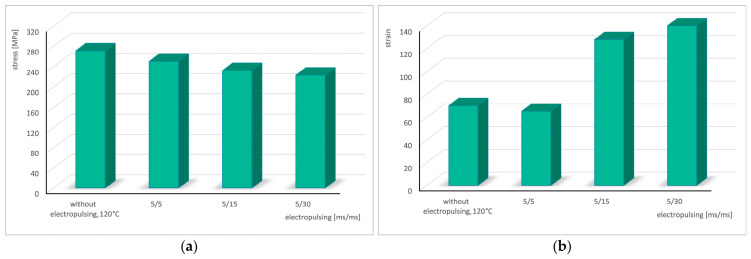
Change in stress (**a**) and strain (**b**) in the cyclic torsion test with the application of electric pulses of duration *t_d_* = 5 ms depending on the pause *t_p_*.

The following bar charts illustrate the variations in stress and strain as a function of the pause time between successive electrical pulses (Figure 10).

**Figure 11:**
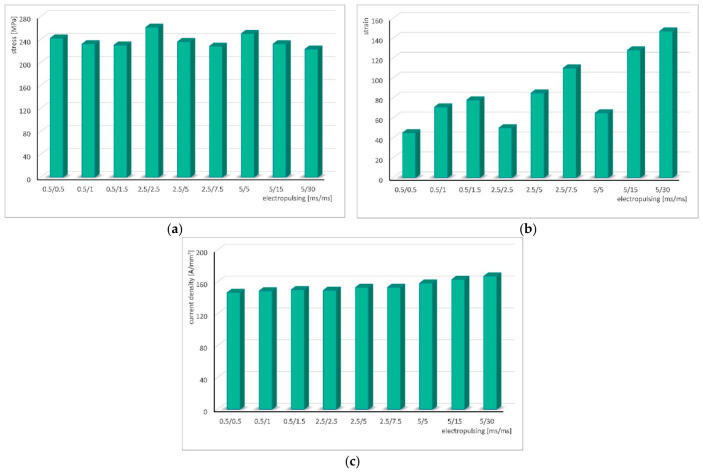
Change in stress (**a**), strain (**b**) and current density depending (**c**) on the electric pulse parameters.

Figure 11 presents bar charts of the changes in stress (Figure 11a), strain (Figure 11b), and current density as a function of the electrical pulse parameters. The current density, with increasing pulse duration and pause time between pulses, generally shows a slight upward trend, although its values remain similar for all applied electrical parameters (Figure 11c).

**Figure 12:**
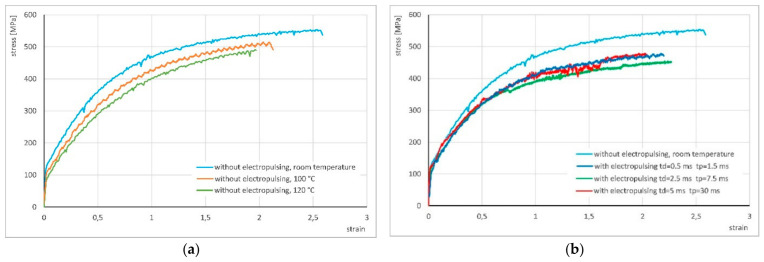
Stress–strain curves for monotonic torsion without current flow (**a**) and with the application of electric pulses (**b**).

The authors also conducted additional investigations to complement the above results. These included conventional monotonic torsion tests at a strain rate of $\dot{\varepsilon }$ = 1 s^−1^ without electric current flow at room temperature as well as at 100 °C and 120 °C. Later, tests were carried out with the application of current pulses at the same strain rate, using pulse durations of *t_d_* = 0.5, 2.5, and 5 ms and pause times of *t_p_* = 3*t_d_*, identical to those applied in the cyclic torsion experiments. The results are presented in Figure 12.

Figure 12a shows a comparison of the stress–strain curves obtained in monotonic torsion tests without electric current flow, conducted at room temperature as well as at 100 °C and 120 °C. The increase in temperature caused a reduction in stress from approximately 550 MPa to 512 MPa at 100 °C and to 492 MPa at 120 °C.

Figure 12b presents the stress–strain curves from monotonic tests with the application of current pulses in comparison to the reference curve obtained at room temperature without current flow. Similarly to the cyclic tests, the electric current flow and the associated temperature rise in the material resulted in a greater reduction in stress than the temperature increase produced by externally heating the specimen. Compared with monotonic tests performed at elevated temperatures (without current flow), the stress in the electrically assisted tests decreased by an average of approximately 100 MPa. It is also worth noting that, regardless of the electrical parameters used (0.5/1.5 ms, 2.5/7.5 ms, and 5/15 ms), the curves exhibit very similar shapes, with no significant differences in stress or strain values between them.

## 4. Discussion

The results indicate that, in pulsed current with an isophase characteristic (*t_d_* = *t_p_*), a pause between successive pulses equal to the pulse duration is too short, causing the effects to become similar to those observed under the application of continuous current during deformation.

Pulsed electric current—that is, current flowing intermittently, with an amplitude that changes very rapidly and periodically over time—is characterized by a very strong electrical stimulus that affects the microstructure and material properties of metals. Compared with continuous current, millisecond- or microsecond-scale electrical pulses can provide enhanced EPE through the instantaneous and localized activation of dislocation rearrangement and potentially induced recrystallization, thereby increasing the material’s deformability, as demonstrated in previous studies on electrically assisted tensile deformation [25,26,27,28,29,30,31].

As mentioned above, the authors believe that isophase current leads to similar effects as continuous current. The application of continuous current during deformation leads to a significant reduction in stress. However, unlike pulsed current, the flow of continuous current can cause a decrease in elongation, as observed, for example, in [31]. When applying continuous current, much lower current densities should be employed; this is due to the rapid heating of the material through which the continuous current flows, even at low densities. A large increase in temperature leads to rapid heat accumulation that exceeds the stress relaxation rate, resulting in the premature failure of the material [30].

The application of current pulses allows for the utilization of—in addition to Joule heating—the athermal effects of current flow, which are independent of temperature. These effects also contribute to increased plasticity, albeit to a lesser extent. In contrast, in the case of continuous current, the primary mechanism affecting the material is Joule heating, i.e., the thermal effect.

Pulsed current enables enhanced plasticity by dynamically influencing dislocation motion while simultaneously mitigating the detrimental effects of excessive heating. Due to its nature, continuous current often results in purely thermal effects, which in many cases are less effective at facilitating the plastic flow of metal than dynamic pulsed interactions.

In contrast to pulsed currents, the application of continuous current often results in lower strain and inferior results in plasticity tests. This stems from the fact that the athermal effect (the so-called pure electroplastic effect) occurs during the pulse flow, whereas the interval between successive pulses serves a purely thermodynamic role. The key to achieving athermal effects is ensuring conditions in which Joule heating does not dominate over kinetic effects. The primary purpose of the inter-pulse intervals is to dissipate the heat generated during the pulse itself (Joule heating) before the next pulse occurs. If the interval is too short, heat accumulates within the material, raising its bulk or local temperature. In such cases, the pulsed current begins to yield effects similar to those of the continuous current. Therefore, the interval between pulses should be long enough for the specimen temperature to drop to a level where athermal phenomena can manifest. Meanwhile, isophase current is characterized by a 1:1 ratio between pulse duration and interval. The thermal stress relaxation time depends, among other factors, on the specimen dimensions and the thermal diffusivity of the material under study. The inter-pulse interval should be calibrated to this relaxation time. This leads to the conclusion that to induce increased deformation, an appropriate interval between pulses is necessary, which must be longer than the pulse duration itself.

In the figures presenting the results of torsion tests with the application of electrical pulses of *t_d_* = 2.5 ms and a pause time of *t_p_* = 7.5 ms (Figure 7c), as well as with pulse duration *t_d_* = 5 ms and pause times of *t_p_* = 15 ms and 30 ms (Figure 9b,c)—that is, in experiments with long pauses, three and six times longer than the pulse duration—the shape of the stress–strain curves is noteworthy, as it is characteristic of symmetric high-temperature cyclic torsion. To attempt to explain this behavior, reference must be made to the properties of CuZn30 brass.

The recrystallization temperature of CuZn30 brass is not a strictly defined value but depends on the degree of strain hardening introduced during cold working. The greater the hardening, the lower the temperature required to initiate recrystallization. It also depends on the conditions of the heat treatment process, such as the heating rate and the soaking time. In general, the recrystallization temperature can be estimated using the following equation:

*T_recrystallization_* ≈ (0.35 ÷ 0.60) × *T_melting point_* (5)

Thus, since the solidus temperature of CuZn30 brass is approximately 907 °C, this means that the recrystallization temperature will be on the order of above roughly 315 °C.

**Figure 13:**
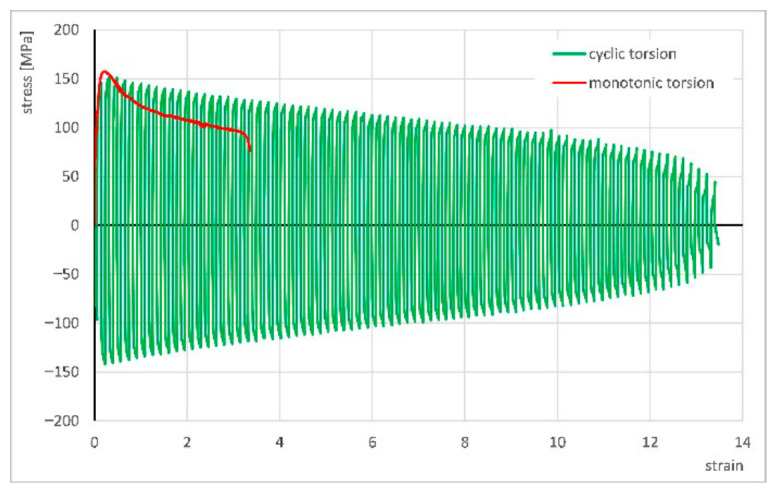
Comparison of stress–strain curves for monotonic torsion at strain rate of $\dot{\varepsilon }$ = 1 s^−1^ and cyclic torsion at strain rate of $\dot{\varepsilon }$ = 1 s^−1^ with strain amplitude of A = 0.06, both at 600 °C [24].

Brass CuZn30, as material with low stacking fault energy, exhibits limited capability for high-temperature dynamic recovery. As the dislocation density increases, the stored energy reaches a level that enables the onset of dynamic recrystallization once the critical strain required to initiate recrystallization is achieved. This leads to material weakening, manifested as a decrease in flow stress within the strain range from the plastic strain corresponding to the maximum flow stress to the strain marking the beginning of steady-state plastic flow [24]. The results of the authors’ previous studies demonstrated that, in the hot cyclic torsion test, after an initial increase in stress, material weakening occurs due to the process of dynamic recrystallization: the stress rapidly reaches maximum value and subsequently decreases gradually. Strengthening takes place only in the initial stage of material deformation. Thereafter, plastic flow appears, although this state is not stabilized because the stress decreases in each successive deformation cycle. Moreover—as established by the authors in their earlier work—cyclic hot torsion produces incomparably lower strains and stresses compared to tests involving the application of electrical pulses [24]. The stress–strain curve for cyclic torsion of CuZn30 at 600 °C, with a strain rate of $\dot{\varepsilon }$ = 1 s^−1^ and a strain amplitude of A = 0.06, is shown in Figure 13.

The α-brasses are characterized by good ductility at room temperature and are capable of undergoing large strains in cold working processes. Alloys with copper content exceeding 63%—such as the CuZn30 investigated here—can be significantly deformed at room temperature and are widely used in the production of various components manufactured through cold forming operations. In the authors’ previous studies on cyclic torsion of CuZn30 at 600 °C (Figure 13), it was shown that the attainable strain is much lower than in analogous tests conducted at room temperature (Figure 3a) [24].

With regard to the shapes of the curves obtained in tests with the application of electrical current pulses, shown in Figure 7c and Figure 9b,c, it should be noted that the thermal imaging camera—used to monitor the temperature of the deforming specimen through which the electric current flows—measures only the surface temperature, not the temperature within the entire volume of the material. Electrically assisted deformation tests cannot measure the temperature inside the material. The internal temperature can only be estimated based on the observed effects that occurred during testing.

At this point, it is worth recalling the basics of issues related to EAF research. The most common theories describing the changes in material behavior occurring during plastic forming processes assisted by electric current flow are divided into thermal and athermal theories. Thermal theories are related to the heat generated during current flow. Athermal theories are associated with other broadly understood phenomena linked to current flow and are referred to as the EPE. The main athermal theories include the electron wind theory, the theory of magnetoplasticity, and the theory of excess free electrons [24]. The most common thermal theories include the theory of bulk Joule heating, and the theory of localized Joule heating [24].

The EPE refers to the intrinsic mechanism through which an electric current significantly alters the mechanical properties of metals. According to the authors, the electron wind theory and both theories regarding Joule heating constitute the primary principles governing the phenomena occurring during EAF. However, the interaction of multiple concurrent phenomena hinders a precise and unambiguous explanation of electron–dislocation interactions [22].

The electron wind theory posits an interaction between electrons and dislocations within a metal through which an electric current flows. Plastic deformation of the material sets dislocations in motion. When the velocity of the electrons flowing through the metal exceeds the velocity of the dislocation movement, and the vectors of both velocities are compatible, the electrons exert a specific force on the dislocations, accelerating them and facilitating their motion; this phenomenon is termed “electron wind”. However, research results in the available literature indicate that the electron wind may account for only a small fraction of the observed changes and should not be considered the primary mechanism of the alterations induced during EAF processes [24].

The bulk Joule heating theory is based on Joule’s law, which states that the amount of heat released during the flow of an electric current through a conductor is directly proportional to the product of the conductor’s resistance, the square of the current intensity, and the duration of its flow. An increase in temperature results in the thermal softening of most metals and their alloys, leading to improved formability. However, it appears that the bulk Joule heating theory alone is insufficient to describe the changes occurring in materials during plastic deformation under current flow. It has been observed that a metal conventionally heated to the temperature recorded during EAF tests, and subsequently deformed at that temperature, behaves differently than the same material deformed in an analogous manner but within an EAF process [24].

The localized Joule heating theory is an extension of the bulk Joule heating theory. It assumes thermal heterogeneity within the material during current-assisted forming. More heat is released in areas of higher resistivity, such as non-metallic inclusions, precipitates, dislocations, stacking faults, and grain boundaries. As is widely known, an increase in temperature enhances dislocation mobility. Such locally concentrated temperature rises—induced by the flow of electric current—can trigger more intensive dislocation movement than conventional heating. Consequently, the thermal softening induced by the current flow is more closely linked to the heterogeneity of the metal’s microstructure. As strain increases, the density of dislocations and other microstructural defects rises, leading to higher resistivity, which, upon the application of current pulses, is responsible for the temperature increase. Subsequently, a greater number of dislocation clusters and a higher amount of stored energy result in an increased tendency for dynamic recrystallization, ultimately enhancing the material’s plasticity. Localized temperature rise is naturally coupled with the volumetric temperature increase of the metallic material; therefore, the localized Joule heating theory cannot be separated from the bulk Joule heating theory described above. Experimental results indicate that these two theories likely provide the best explanation for the changes in material properties occurring during electrically assisted forming processes [24].

The pulsed current passing through the deforming specimen encounters regions that are heavily deformed and contain a high density of dislocations, which leads to a local increase in electrical resistance and may cause significant local temperature rises above the recrystallization temperature. These temperature spikes could not be captured by the thermal camera. Such localized temperature increases promote the nucleation of recrystallization and, consequently, the onset of dynamic recrystallization.

As a result, this may lead to substantial increase in strain. The coexistence of two adjacent regions—a deformed region (prior to recrystallization) and an already recrystallized region—may contribute to very large deformations. This may be related to the fact that unrecrystallized areas exhibit more favorable strain-hardening coefficient, which can enhance the achievable strains. Furthermore, the recrystallized regions are continuously subjected to further deformation, while local recrystallization emerges in new locations, preventing excessive dislocation accumulation that could otherwise lead to crack initiation in the deforming material. Thus, the location of the recrystallizing zone shifts as new, highly deformed regions with increased dislocation density appear. During torsion assisted by electrical current pulses, the deformation and recrystallization processes occurring simultaneously in different regions of the material produce a synergic effect that enables the attainment of such high strain levels.

This is consistent with the aforementioned localized Joule heating theory. Consequently, even if the local temperature rise was insufficient to initiate recrystallization due to being too low, it is widely established that an increase in temperature nonetheless enhances dislocation mobility [32,33]. Such a locally concentrated temperature rise—induced by the flow of electric current—can trigger more intensive dislocation movement than conventional heating. Electrically induced “hot spots” located at grain or phase boundaries act as nucleation sites for new grains or phases [5]. Consequently, the thermal softening induced by the current flow is more closely linked to the heterogeneity of the metal’s microstructure. Indeed, the material under study exhibits such a microstructure when subjected to deformation in cyclic torsion tests [22].

Regarding the results of monotonic (unidirectional) torsion presented in Figure 12, it should be noted that the loading scheme of the material in monotonic tests differs from that in cyclic torsion trials. The conducted monotonic torsion tests revealed a very low sensitivity of the CuZn30 alloy to changes in current parameters. This certainly warrants further experimental investigation, considering factors such as strain rate effects, the initial state of the material, etc., as well as microstructural analysis using TEM or numerical modeling of microstructural evolution through microscale mathematical modeling.

In the present study, the authors did not perform microstructural examinations and acknowledge that the absence of such analysis constitutes a certain limitation. However, EBSD analyses were conducted in a previous study [22] and the results did not yield the expected insights. The microstructure of CuZn30 after cyclic torsion (i.e., after very significant plastic deformation) was so heavily degraded that it was impossible to obtain diffraction patterns of sufficient quality. The EBSD detector cannot resolve such severely distorted microstructures, resulting in poor signal matching, which appears as black areas on the EBSD maps shown in [22]. Therefore, the authors saw no merit in repeating these analyses in the current work, as the EBSD maps would remain largely uninterpretable. In future studies, the authors plan to focus on TEM microstructural investigations to attempt to confirm the proposed mechanisms.

## 5. Conclusions

The authors of this article were the first to initiate research on electrically assisted torsion. Based on the conducted investigations and the analysis of the results, the following primary conclusions were formulated:

1. During electrically assisted torsion, an increase in the temperature of the tested material occurs. For pulses with a duration of *t_d_* = 0.5 ms, only a negligible change in temperature was observed; however, increasing the pulse duration led to a distinct temperature rise.
2. For the first time, it has been demonstrated that torsion assisted by electric current yields better results than torsion conducted without current at a comparable elevated temperature.
3. The application of current pulses with the described parameters results, in all cases, in a reduction in stress compared to tests without current flow conducted under analogous conditions.
4. When current pulses with the shortest durations are applied (0.5 ms), the stress reduction remains practically constant, regardless of the pause duration. At longer pulse durations (2.5 and 5 ms), the pause length between pulses has a greater effect on the stress level.
5. When an isophase current is applied (*t_d_* = *t_p_*), it has been demonstrated for the first time that torsion assisted by such a current not only fails to improve the strain but actually leads to its deterioration. For the 0.5/0.5 ms current, the strain decreased by 21.05%; for 2.5/2.5 ms, the strain decreased by 20.63%; and for 5/5 ms, the reduction amounted to 7.14%. This leads to the conclusion that achieving strain improvement requires an appropriate pause time *t_p_* between pulses, which must be longer than the pulse duration *t_d_*.
6. In monotonic torsion assisted by electric current flow, CuZn30 brass showed very low sensitivity to changes in current parameters.
7. Selected experimental results indicate that it is possible to choose such process parameters that lead to a reduction in stress and/or a significant increase in strain. This is important for designing energy-efficient metal-forming processes that require lower deformation forces and enable higher achievable strains.
8. Future research directions could include, for example, systematic variation of electrical pulse parameters, microstructural studies TEM, modeling, and integration with data-driven and machine learning methods, which the authors have already planned for their further work on electrically assisted deformation.

## Figures and Tables

**Table 1:** Stress and strain results obtained during cyclic torsion without current flow at room temperature, 100 °C and 120 °C.

Test Conditions	Stress [MPa]	Strain
without electropulsing at room temperature	306	57
without electropulsing at 100 °C	285	63
without electropulsing at 120 °C	271	70

**Table 2:** Stress and strain results obtained during cyclic torsion without current flow at room temperature and with the application of electrical pulses with duration *t_d_* = 0.5 ms and pause times *t_p_* = 0.5 ms, 1 ms and 1.5 ms.

Test Conditions	Stress [MPa]	Change in Stress [%]	Strain	Change in Strain [%]
without electropulsing at room temperature	306	-	57	-
with electropulsing 0.5/0.5 ms (isophase)	242	–20.91	45	–21.05
with electropulsing 0.5/1 ms	232	–24.18	71	+24.56
with electropulsing 0.5/1.5 ms	230	–24.84	78	+36.84

**Table 3:** Stress and strain results obtained during cyclic torsion without current flow at 100 °C and with the application of electrical pulses with duration *t_d_* = 2.5 ms and pause times *t_p_* = 2.5 ms, 5 ms and 7.5 ms.

Test Conditions	Stress [MPa]	Change in Stress [%]	Strain	Change in Strain [%]
without electropulsing at 100 °C	285	-	63	-
with electropulsing 2.5/2.5 ms (isophase)	261	–8.42	50	–20.63
with electropulsing 2.5/5 ms	236	–17.19	85	+34.92
with electropulsing 2.5/7.5 ms	228	–20.00	110	+74.60

**Table 4:** Stress and strain results obtained during cyclic torsion without current flow at 120 °C and with the application of electrical pulses with duration *t_d_* = 5 ms and pause times *t_p_* = 5 ms, 15 ms and 30 ms.

Test Conditions	Stress [MPa]	Change in Stress [%]	Strain	Change in Strain [%]
without electropulsing at 120 °C	271	-	70	-
with electropulsing 5/5 ms (isophase)	250	–7.75	65	–7.14
with electropulsing 5/15 ms	232	–14.39	128	+82.86
with electropulsing 5/30 ms	223	–17.71	147	+110.00

## Data Availability

The original contributions presented in this study are included in the article. Further inquiries can be directed to the corresponding author.
